# The Association between Tea Consumption and Nasopharyngeal Cancer: A Systematic Review and Meta-Analysis

**DOI:** 10.31557/APJCP.2020.21.8.2183

**Published:** 2020-08

**Authors:** Simon I Okekpa, Rabiatul Basria S. M. N. Mydin, Sivaraj Ganeson, Saravanackumar Gopalan, Muhamad Yusri Musa

**Affiliations:** 1 *Oncological and Radiological Sciences Cluster, Advanced Medical and Dental Institute, Universiti Sains Malaysia, 13200 Bertam, Kepala Batas, Pulau Pinang Malaysia. *; 2 *Department of Medical Laboratory Science, Faculty of Health Sciences, Ebonyi State University, Abakaliki, 840001 Ebonyi state, Nigeria.*; 3 *School of Distance Education, Universiti Sains Malaysia, Penang, Malaysia. *; 4 *Department of Biological Sciences, National University of Singapore, 14 Science Drive 4, 117543 Singapore. *

**Keywords:** Nasopharyngeal cancer, tea consumption, risk factors of nasopharyngeal cancer

## Abstract

**Introduction:**

Heated debates have been on-going about tea consumption and the incidence of cancer, especially in head and neck cancer types. This study aimed to review the association between tea consumption habits and nasopharyngeal cancer (NPC).

**Methods:**

This review was carried out in accordance with the PRISMA-P protocol. Literature search for journal articles that published studies on the relationship between tea consumption and NPC was performed via databases, such as Elsevier, PubMed, Science Direct, Springer Link, Google, and Google Scholar, for 10 years from 2008 to 2018. Relevant studies were obtained by applying the pre-determined keywords, such as nasopharyngeal cancer, tea consumption and NPC, risk factors of NPC and benefits of tea consumption.

**Results:**

A total of 126 articles was retrieved. These articles were subjected to eligibility assessment. Six articles remained after applying the inclusion criteria. Results suggest that habitual tea consumption reduces NPC. Tea consumption significantly reduces NPC with all the studies having a p-value ≤0.05. Meta-analysis showed statistical association between tea consumption and NPC risk with OR=0.865 at 95% CI (0.806-0.929).

**Conclusion:**

This study suggests that habitual tea consumption could be associated with prevention of NPC development. Additional studies are needed to further understand the molecular role of bioactive compound and potential health benefit of tea consumption in NPC prevention.

## Introduction

Tea is obtained from the leaves of Camellia sinensis plant and frequently consumed due to its flavour and potential health benefits (Goldbohm et al., 1996). Tea has seven classifications based on brewing method. The classifications are ‘white’, ‘yellow’, ‘green’ (which is a non-fermented tea), ‘oolong’ (incompletely fermented tea), ‘black’ (fully fermented tea), ‘aged pu-erh’ (extremely fermented aged tea) and ‘mature pu-erh tea’ (Yi et al., 2015; Koch et al., 2018). The global consumption rate of different tea types varied. The pu-erh and oolong tea types were most commonly consumed tea in China, black tea in Europe and America and green tea in East and Southeast Asia (Lin et al., 2003; Crespy and Williamson, 2004; Ng et al .,2014). Habitual tea consumption has been associated with prevention of various cancers (Lambert and Yang, 2003). The bioactive contents of tea, such as phenols and polyphenols, inhibit the growth of cancer cells (Ruan et al., 2010). However, the potential health benefit of tea consumption in NPC prevention in the context of habitual patterns is not clear. Therefore, this study determined the association between tea consumption and NPC.

## Materials and Methods

*Methodology*

*Search strategy*

**Figure 1:**
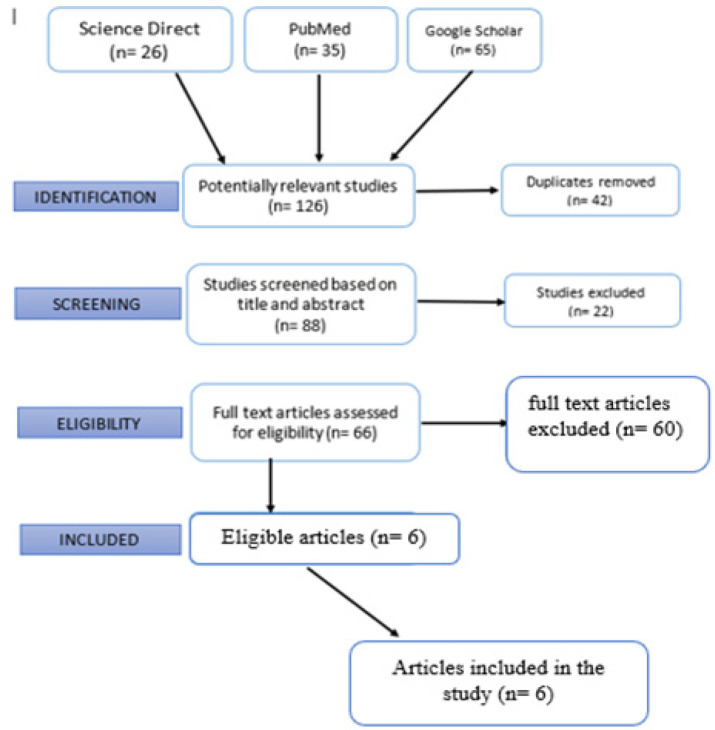
Flowchart for Literature Search. A total of 126 publications were retrieved from the initial literature search, namely, 26 articles from Science Direct, 35 articles from PubMed and 65 articles from Google Scholar on the basis of the flowchart presented for study selection. A total of 42 duplicate articles were excluded, and 88 articles were screened on the basis of the title and abstract. Among the 88 articles, a total of 66 full-text articles were assessed for eligibility after excluding 22 articles that did not meet the research criteria. The excluded articles were categorised as reviews, case reports and unrelated articles. A total of 60 full-text articles were further excluded, and the remaining 6 articles were finally deemed eligible in terms of data on the association between tea consumption and NPC cancer.

This review was conducted in accordance with PRISMA-P protocol. It began with Comprehensive literature search from electronic databases; Science Direct, PubMed, and Springer Link was carried out. General search engine such as Google Scholar was also used to obtain relevant articles(Fig.1). Keywords used for the literature search were: general components of tea, Tea consumption & Nasopharyngeal Cancer (NPC), Risk factors of Nasopharyngeal Cancer (NPC), Benefits of Tea consumption, Risk Factors of Tea consumption, Green Tea and Black Tea. Manual search for relevant references cited in the selected articles was performed to obtain additional relevant studies.

*Selection criteria*

Eligibility and relevance of the selected articles were carried out. The inclusion criteria for this systematic review are; all the selected publications must fall within the past 10 years (2008 till 2018), it must be studies that involves only NPC cases, the paper must address the association of tea consumption and NPC risk, and must posses case-control design. The criteria for exclusion are: all publications before 2008, studies involving other types of head and neck cancer (HNC) other than NPC and other cancer types, and studies not addressing the association of tea consumption with NPC risk.

*Data extraction*

The data extraction were conducted using a standardized data extraction form, extracting information such as the name of first author, the year of publication, study design, sample size, number of cases and control, study size, gender of participants, type of the tea, tea drinking definitions, NPC definitions, its risks, and the OR value with 95% CI (Chen and Long, 2014). The above extraction criteria used were aimed at providing quality evidence and eliminating biases. Guideline and checklist applied in the study criteria is Preferred Reporting Items for Systematic Review and Meta-Analysis Protocols (PRISMA-P).

*Data analysis*

The data obtained estimated the OR value in this analysis because of increasing rate of nasopharyngeal cancer. The ORs and 95% CIs of all included studies were obtained using variance-based method with a fixed effect. Descriptive analysis of tea consumption in respect to nasopharyngeal cancer risk, dose response relationships in terms of daily or monthly and lifetime tea consumption were carried out. All the included articles collected their information from either Medical record and/or questionnaire to observe the effect of tea consumption. All the obtained information was tabulated.

*Statistical analysis*

Meta-analysis was performed for tea consumption using the case-control studies that reported association tea consumption with NPC. Odds ratio for each of the selected studies were individually computed from the cases and controls and summarized with fixed-effect model. Statistical heterogeneity of the included studies was evaluated in the meta-analysis through Q and I^2^. Forest plot for tea consumption was also computed, using comprehensive meta-analysis. Estimation of potential publication bias was estimated using the funnel plot, which plotted standard error of each study against its OR. Asymmetrical plot indicates publication bias. All statistical analyses were carried out using comprehensive meta-analysis Software. A P-value of <0.05 indicates statistically significant.

## Results

*Effects of tea consumption habit*

All the articles included in this study, reported that tea consumption (overall all type of tea) could help in cancer prevention. All the studies were case-control studies (Table 1). It also reavealed that, tea consumption habit contributes to the effect of the tea on the patient.

*Tea consumption and nasopharyngeal cancer*

The overall analysis of all the six studies, including the case control and cohort studies, found that tea drinking was associated with significant reverse in NPC with all having p-value of ≤ 0.05 (Table 2).

*Meta-analysis for tea consumption*

**Figure 2:**
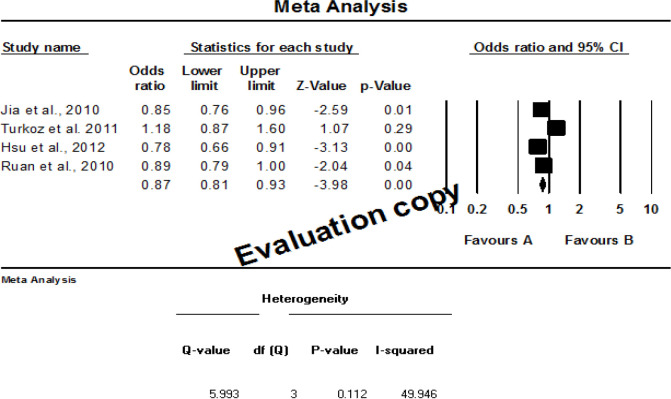
Forest Plot for Association of Tea Consumption with NPC. The Individual black box on the forest plot represents a single study indicating the result plotted whereas the lines shows the 95% confidence interval of the results displayed. The bigger black box represents larger sized study. The diamond shaped box at the lowest part of the forest plot indicates the mean of the results when all the studies were combined (0.865) whereas the horizontal points in the diamond shaped box represents the 95% confidence intervals (0.806; 0.929). The vertical line was not crossed by the horizontal points of the diamond shaped box, therefore, this review observed statistical variation amongst the studies. I^2^ statistic showed low heterogeneity (I^2^=49.95%; P=0.11) of the included studies, indicating consistency. Note that Inconsistent studies is indicated by I^2^ value of >50%.

**Figure 3:**
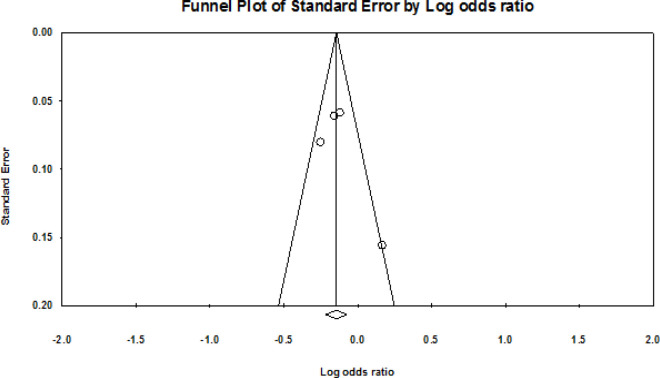
Funnel Plot for Tea Consumption and NPC. Each dot indicates individual study. The y-axis represents standard error whereas odds ratio of the study is represented by X-axis. Larger studies with higher power are represented by the dots close to the top of the funnel whereas, various standard errors are represented by the plot shape and dot scatter. All the studies do not have same standard error sizes that is why all the dots did not drop on the horizontal line

The meta-analyses results for tea consumption (tea drinkers versus non-tea drinkers) are shown in Figure 2 and 3. The forest plot (Figure 2) of the meta-analysis showed statistical association between tea consumption and NPC risk with OR=0.865; 95% CI=0.806-0.929) and low statistical heterogeneity (I^2^=49.95%; P<0.00). Meta-regression-analysis was not conducted to investigate potential factors that could influence the odds ratio summaryof the lowered NPC risk due to variation in study factors and characteristics. Funnel plot, showed indicates no visual publication bias (Figure 3). 

**Table 1:** Association between Tea Consumption Frequencies and Nasopharyngeal Cancer

Sources	Study participant	Data collection method & location	Outcome measured	Adjusted OR estimates (95% CI)
Jia et al., 2010	425 cases and	medical records	Herbal tea habit	Monthly: OR = 0.46(0.38-0.56)
	717 controls,	China.		
	369 cases			Weekly: OR = 0.84(0.68-1.03)
	347 controls			
	439 cases			Less than a month 1.0
	347controls			
Turkoz et al., 2011	10patients, 37 controls,	Questionnaire Interview Turkey	Tea in general	Never: OR = 1,0
	149 patients, 130 controls,			<10 glass/day: OR = 5.55 (2.16-14.24)
	24patients, 16 controls			10 glass/day or more: OR = 1.31(0.67-2.57)
Hsu et al., 2012	263 cases,	Questionnaire	Black tea	(0 times/week): 1.00 (referent)
	193controls	Taipei city		
	53 cases			(0.5 times/week): 0.66 (0.43–1.03)
	61 controls			
	54 cases			(≥0.5times/week): 0.69 (0.44–1.08)
	66 controls			
	268 cases,		Green tea	(0 times/week): 1.00 (referent)
	194controls	Questionnaire Taipei city		
	36 cases			(˂1 times/week): 0.58 (0.35–0.98) *
	45 controls			
	64 cases			(≥1times/week): 0.61 (0.40–0.91) *
	78 controls			
	152 cases,		Oolong tea	(0 times/week): 1.00 (referent)
	100controls	Questionnaire Taipei city		
	108 cases			(1-3 times/week0.70 (0.47–1.03)
	113 controls			
	107 cases			(˃3times/week): 0.66 (0.44–0.98)
	107 controls			
Ruan et al., 2010	903 cases	Questionnaire,	Tea in general	0.62(0.52–0.74)
	1095controls	Interview. China		

OR, Odds Ratio; CI, Confidence Interval

**Table 2:** Association of Tea Consumption with NPC Prevention

Types of tea	Study	*P*-value
Green	Hsu et al., 2012	<0.05*
Black	Hsu et al., 2012	<0.05*
Oolong tea	Hsu et al., 2012	<0.05*
Herbal tea	Jia et al., 2010	<0.001**
	Xie et al., 2015	<0.012**
	He et al 2015	< 0.001**
Tea in general	He et al 2015	< 0.001**
	Turkoz et al. 2011	<0.001**
	Ruan et al., 2010	<0.001**

## Discussion

This review revealed inverse correlations between consumption frequencies of tea and NPC risk (Table I), with each study having an odds ratio of less than 1 (1 is the reference value). Khan and Mukhtar (2013) reported that tea consumption has dose–response relationships with frequency, duration and concentration (Khan and Mukhtar, 2013). Moreover, Koch et al., (2018) suggests that certain adult dietary patterns might have protective effects against the development of NPC. The frequency of tea consumption could possibly associated with reduction in NPC risk.

Furthermore, meta-analysis data suggest that tea consumption was associated to NPC risk reduction with fixed effect model score showing OR=0.865 at 95% CI=0.806-0.929 and a low statistical heterogeneity I2=49.95%; P<0.00 (Fig.2). However, this current meta-analysis is limited by disparity in the parameters studied by our data source. This investigation found a contrary association between NPC risk and tea consumption from a previous report by Lin et al (2019), which revealed that the risk of NPC was increased among people who consume herbal tea for up to five years compared to those who cosumed less. Therefore, few studies have reported that, inconsistent habit of tea drinking could lead to NPC risk.

In regards of types of tea consumption and NPC, this study also found all ttypes of tea consumption significantly contribute in NPC prevention with p-value of ≤ 0.05 (Table II). Lin et al. (2012) and Fujiki et al. (2018) explained the molecular action of catechins that inhibit cell growth and induce apoptosis in parental NPC cells. Previous studies also discovered that tea such as green tea and Oolong tea have protective effects on NPC risk, which is consistent in other studies concerning other types of cancers (Qihua et al., 2008; Tang et al., 2009; Lee et al., 2010; Ogunleye et al., 2010). Therefore, strong correlation was observed between habitual consumption of all tea types and NPC risk reduction.

The cancer-preventive effect of tea could be attributed to the presence of antioxidant and polyphenols, such as catechins, the aflavins, EGCG, ECG and thearubigins, which contribute to the health benefits of tea. Valenzuela, (2004) reported that EGCG and ECG can inhibit the activities of acetylase, kinase and methylase, thus determining tumour developmental processes (Valenzuela,2004). Luo et al., (2014) reported that EGCG effectively inhibited the migration and invasion of cancer cells, as well as decreasing the metastasis of cancers (Martínez et al., 2018; Luo, et al 2014). Thefore, bioactive contents of tea, such as phenols and polyphenols, could possibly inhibit the growth of cancer cells and protect againts cancers.
